# Rocket Dynamics of Capped Nanotubes: A Molecular Dynamics Study

**DOI:** 10.3390/nano14131134

**Published:** 2024-06-30

**Authors:** Mustafa S. Hamad, Matteo Morciano, Matteo Fasano

**Affiliations:** Department of Energy, Politecnico di Torino, Corso Duca degli Abruzzi 24, 10129 Torino, Italy; mustafa.hamad@studenti.polito.it (M.S.H.); matteo.morciano@polito.it (M.M.)

**Keywords:** carbon nanotube, water, nanomotion, molecular dynamics, heat transfer

## Abstract

The study of nanoparticle motion has fundamental relevance in a wide range of nanotechnology-based fields. Molecular dynamics simulations offer a powerful tool to elucidate the dynamics of complex systems and derive theoretical models that facilitate the invention and optimization of novel devices. This research contributes to this ongoing effort by investigating the motion of one-end capped carbon nanotubes within an aqueous environment through extensive molecular dynamics simulations. By exposing the carbon nanotubes to localized heating, propelled motion with velocities reaching up to ≈0.08 nm ps^−1^ was observed. Through systematic exploration of various parameters such as temperature, nanotube diameter, and size, we were able to elucidate the underlying mechanisms driving propulsion. Our findings demonstrate that the propulsive motion predominantly arises from a rocket-like mechanism facilitated by the progressive evaporation of water molecules entrapped within the carbon nanotube. Therefore, this study focuses on the complex interplay between nanoscale geometry, environmental conditions, and propulsion mechanisms in capped nanotubes, providing relevant insights into the design and optimization of nanoscale propulsion systems with various applications in nanotechnology and beyond.

## 1. Introduction

The ability to drive nanoparticles in a supervised way, inspired by the natural self-assembled molecular motors within cellular organisms, is fundamental to the development of nano-sized engines [1] with unique properties. The potential applications of such nanoparticle movements span various domains, including biomedical engineering [2,3] for targeted drug delivery [4], therapeutics [5], tissue engineering and biosensing applications, energy sector applications for fuel production through advanced and enhanced electrolysis [6], and the wider field of automation [7,8]. Various nanoparticle propulsion methodologies have been explored, and are broadly classified as either chemical or physical based on the source of energy they utilize.

Among the various innovative approaches driven by chemical processes to generate nanomotion, the one proposed by Cha and co-workers [9] is of considerable interest. They designed a DNAzyme-based motor which leverages the chemical energy of RNA molecules bound to a carbon nanotube (CNT) to achieve unidirectional motion [9]. Wu and co-workers [10] proposed a method for controlling motion in catalytic polymer-based micro-engines via near-infrared laser irradiation. The working principle of their multi-layered polymer rocket was based on the dissociation of hydrogen peroxide by platinum atoms using thermal energy as a catalyst.

In addition, a variety of strategies driven by physical processes have been investigated. In 2007, Gong and co-workers [11] proposed a molecular water pump design based on water–charge interactions. Their findings were supported by extensive Molecular Dynamics (MD) simulations. The basic concept involved the use of a combination of charges positioned adjacent to a nanopore, and was inspired by the structure of cell membrane channels that conduct water in and out of cells (i.e., aquaporins). The pumping ability has been attributed to the charge dipole-induced ordering of water confined in nanochannels [12,13,14], where water can be easily guided by external fields in a targeted way without osmotic pressure or hydrostatic pressure gradient, contrary to traditional methods of water transport. The proposed concept may offer a new mechanism characterized by simplified design and operation. On the other hand, Ghosh and co-workers [15] detailed the construction and operation of a nanostructured propeller driven by a homogeneous magnetic field. Regan and co-workers [16] constructed and operated a nanoscale linear motor powered by the growth and shrinkage of a nanocrystal under the effect of a low level electrical voltage [16]. Interestingly, a molecular linear motor consisting of CNTs has been investigated by Somada and co-workers [17]. The linear and rotational motion was attributed to the thermal excitation of the CNTs provided by transmission electron microscopy. Moreover, novel designs include the thermoresponsive polymer-based nanomachine designed by Alaghemandi and co-workers [18]. In detail, this nanoengine was based on endgrafted Poly(N-isopropylacrylamide) (PNIPAM) on graphene-like sheets and consisted of a water-filled slab and four PNIPAM chains, which were grafted at one end to one of the slab walls and on the other to a mobile square graphene piston. The basis of the reciprocating motion of the piston was the reversible coil-to-globule transition of polymer chains when changing the temperature of the aqueous environment. More recently, Fasano and co-workers [19] proposed a double-walled CNT-based nanorocket (DWCNT) activated by heating the water confined within the DWCNT. In addition to molecular dynamics simulations, the authors developed an analytical model based on damping forces and Van der Waals interactions that successfully reflected molecular dynamics data. Similarly, Delogu [20] proved by simulations that a capsule-like CNT having two attached electrical charges enclosed within a bigger CNT with one end capped and the second end pored can be propelled in liquid argon via an applied electric field. The phase change of argon from liquid to gas acts as an effective propellant of the model. To further illustrate and highlight the diversity of approaches in achieving nanoparticle motion and the potential of these nanoscale systems, it is worth mentioning the research of Kang et al. [21], who suggested the use of a gas flow to rotate a CNT-based motor; the experimental and numerical research of Barreiro et al. [22], who proposed a temperature gradient-driven cargo transport on CNTs; the numerical study of Dai et al. [23], who studied the motion of neutral CNTs or fullerenes driven by charging the outer housing CNT via quantum mechanical molecular dynamics; and the numerous other approaches involving the thermophoretic effect [24,25,26].

Despite the plethora of studies, fabricating nanomotion devices with complex geometries and effectively managing their movement remain challenging tasks. Further studies at different scales are required in order to more thoroughly investigate phenomena that might be involved in the propulsion process, i.e., thermophoresis, vibrational interactions [27,28], interfacial heat exchange [29,30], nanoscale friction [31], phase changes of water at the nanoscale [32,33,34], and especially the effects of operating conditions and geometry on dynamics. Here, we propose a novel nanorocket design in which propulsion is accomplished through photothermal excitation of a coating (e.g., gold) deposited on the walls of a one-end capped CNT which is immersed in water [35,36]. The heated one-end-capped CNT is prone to asymmetric forces generated by the evaporation of water molecules exiting through the open end. Thus, our rationale relies on exploiting evaporated water as a nanorocket propellant. Molecular dynamics simulations are performed to comprehensively study the dynamics of the system. In perspective, we believe that this exploration may suggest new lines of research and promising applications in the field of nanomotion.

## 2. Methods

### 2.1. Nanorocket Concept

**Figure 1:**
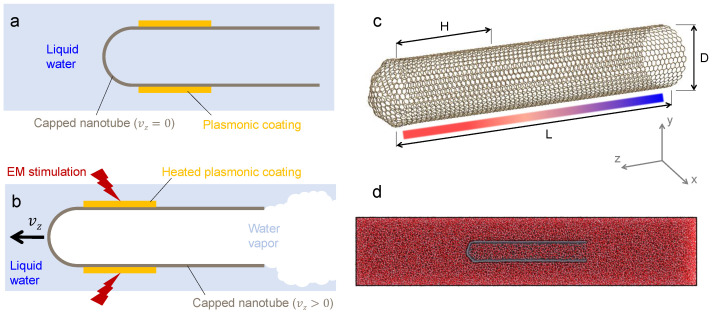
(**a**) Representative schematic of the studied system, showing the one-end-capped CNT with plasmonic coating fully immersed in water. (**b**) Near-infrared heating of the gold coating, water vapor generation, and resultant working principle. (**c**) A 3D view of the CNT with respect to the reference frame in GROMACS. *H* represents the length of the plasmonic coating mimicked in the MD simulations; the color bar qualitatively represents the temperature gradient along the capped carbon nanotube, with higher temperatures depicted in red. (**d**) Rendering of one of the simulated molecular dynamics domains (the mid-plane section of setup 6; cf. Table 1). Water molecules are represented in red and carbon atoms in gray.

The nanorocket concept proposed herein consists of a CNT with one capped end and a second open end. Furthermore, a gold coating is envisaged to have been chemically deposited on the walls [37,38,39]. This system requires immersion in water. By irradiating the nanorocket with near-infrared radiation, the temperature of the gold coating is expected to reach up to 1300 K [35,36], causing the evaporation of neighboring water molecules. Water molecules then exchange momentum with the walls of the CNT and flow out of the CNT through the open end, resulting in significant propulsion. Simple schematics showing a typical configuration and the working principle are depicted in Figure 1a,b, respectively, along with the nanoscale structure of a simulated CNT in Figure 1c.

### 2.2. Simulation Details

The nanorocket dynamics were investigated by performing molecular dynamics simulations using the open-source GROMACS 4.6.5 package [40].

Bonded interactions between carbon–carbon and oxygen–hydrogen were modeled by harmonic potentials. Stretching of covalent bonds, bond angle deformations, and proper dihedral deformations were considered. In detail, the bond stretching between two covalently bonded atoms (i.e., *i* and *j*) is represented by the following harmonic potential:

(1) $$V_{b}\left(r_{ij}\right)=\frac{1}{2}k_{ij}^{b}{\left(r_{ij}-b_{ij}\right)}^{2}$$

where $V_{b}$ is the bond stretching potential, $r_{ij}$ is the distance between two atoms, $k_{ij}^{b}$ is the effective stiffness of the covalent bond (i.e., the harmonic constant), and $b_{ij}$ is the equilibrium distance. Then, the bond-angle between a triplet of bonded atoms (i.e., *i*, *j*, *k*), represented by a harmonic potential, is in the following form:

(2) $$V_{a}\left(\theta_{ijk}\right)=\frac{1}{2}k_{ijk}^{\theta }{\left(\cos\theta_{ijk}-\cos\theta_{ijk}^{0}\right)}^{2}$$

where $V_{a}\left(\theta_{ijk}\right)$ is the bond-angle potential, $k_{ijk}^{\theta }$ is the effective bending stiffness of the bond angle, $\theta_{ijk}$ is the bond angular deformation (i.e., the angle between vectors $\vec{r_{ij}}=\vec{r_{j}}-\vec{r_{i}}$ and $\vec{r_{kj}}=\vec{r_{j}}-\vec{r_{k}}$), and $\theta_{ijk}^{0}$ is the equilibrium angle of the bond. Finally, the torsion energy can be evaluated by the following function:

(3) $$V_{d}\left(\xi_{ijkl}\right)=\frac{1}{2}k_{\xi }\left(1-\cos2\xi_{ijkl}\right)$$

where $V_{d}\left(\xi_{ijkl}\right)$ is the proper dihedral potential for four bonded atoms *i*, *j*, *k*, and *l*, while $k_{\xi }$ is the effective torsional stiffness, and $\xi_{ijkl}$ is the dihedral deformation (i.e., the angle between planes ijk and ijl).

Then, non-bonded interactions comprising electrostatic and Van der Waals interactions [41] were considered. The Lennard-Jones potential represents carbon–carbon and carbon–water Van der Waals interactions [42]:

(4) $$U_{LJ}\left(r_{ij}\right)=4\varepsilon_{ij}\left[{\left(\frac{\sigma_{ij}}{r_{ij}}\right)}^{12}-{\left(\frac{\sigma_{ij}}{r_{ij}}\right)}^{6}\right]$$

where $U_{LJ}\left(r_{ij}\right)$ is the Lennard-Jones potential, $\varepsilon_{ij}$ is the depth of the potential well, and $\sigma_{ij}$ is the Van der Waals radius, namely, the finite distance at which inter-particle potential equals to 0. A cut-off distance of 1.2 nm and Lorentz Berthelot combination rules for cross-interaction terms were considered for the Lennard-Jones potential. Both bonded and non-bonded force-field parameters were taken from [43] (see Table 1 there), which have previously demonstrated good prediction capability for the heat and mass transfer behavior of solvated CNTs [19,42,44,45].

The SPC/E model was adopted to describe the structure of the water molecules, with bonded interactions treated using the SETTLE algorithm [46]. This model accurately represents the dynamics and behavior of water [47]. All CNT geometries were produced using NanoCAP 1.0b15 software [48,49]. First, energy minimization of the dry structure was performed. Then, water was added to the system via the *genbox* command by GROMACS (see Figure 1d) and energy minimization of the new system was performed. An NVT simulation was conducted until the steady state was achieved. Velocities were initialized assuming a Maxwell–Boltzmann distribution, then rescaled using the velocity-rescaling thermostat at a temperature of 300 K [50]. The initialization of the pressure of the system was implemented by running an NPT simulation until temperature and pressure achieved equilibrium. A combination of the Nose–Hoover thermostat [51] at 300 K and the Parrinello–Rahman barostat [52,53] at 1 bar was implemented. During the NVT and NPT simulations, harmonic constraints were imposed on the CNT to ensure its positioning at the center of the simulation box during equilibration. The Leap-Frog integrator [54] was used to solve the equations of motion.

For production runs, the same combination of Nose–Hoover thermostat and Parrinello–Rahman barostat was used. A thermostat at *T* temperature (see Table 1) was applied to the carbon atoms mimicking the presence of the gold coating between the closed end and half of the CNT (i.e., H=L/2; see Figure 1c). The remaining unheated carbon atoms and water molecules were allowed to relax to equilibrium. All production simulations lasted for 200 ps, and the target rocket dynamics was completed after the first 150 ps of simulation time (see Supplementary Video S1). A time step of 1 fs was used. To assess the box-size independence of MD simulations, the CNT with 2.5 nm diameter was simulated with water boxes of increasing size and the results were checked for convergence. Moreover, the CNTs analyzed in this study exhibit armchair chirality (excluding the cap), characterized by equal chiral indices (i.e., n = m) [55]. The latter indicates the way in which the graphene sheet is rolled up to form the nanotube; these are reported in Table 1. For clarity and compactness, the list of simulation settings and parameters for all the production runs is reported in Table 1.

## 3. Results and Discussions

### 3.1. Molecular Dynamics Simulation

**Figure 2:**
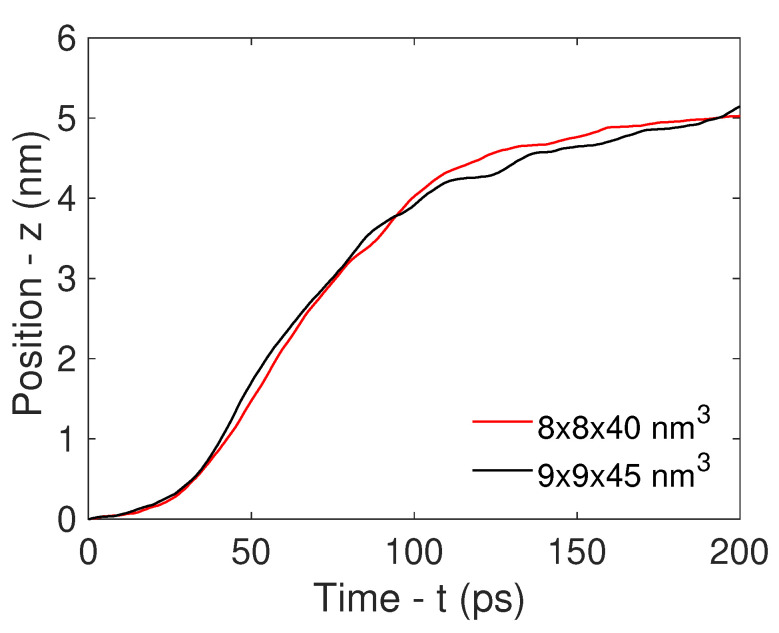
Convergence analysis based on the dimensions of the computational box. The center-of-mass position of the CNT versus time is reported for two boxes with different sizes, namely, 8 × 8 × 40 nm^3^ (red line) and 9 × 9 × 45 nm^3^ (black line).

A first study was performed to check the possible effects of changing the box size on the nanotube dynamics. Indeed, the size of the box should ensure that sufficient water molecules surround the CNT, thereby approaching the real-life physical experiment. The results of this size-independence analysis are reported in Figure 2, and refer to cases in which a CNT with a capped end, a diameter of 2.5 nm, and a length of 15.7 nm was traced in 8 × 8 × 40 nm^3^ (red line, setup 4) and 9 × 9 × 45 nm^3^ (black line, setup 6) water boxes, showing the same dynamics in both cases.

**Figure 3:**
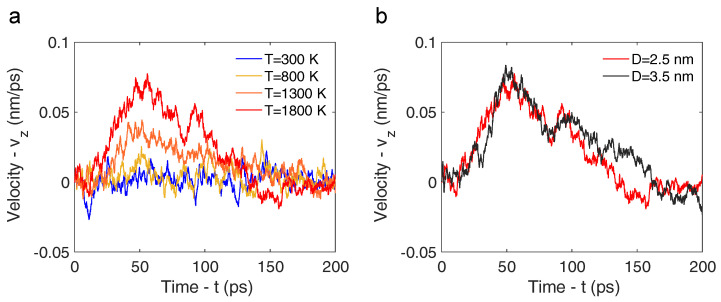
(**a**) Velocity of the center of mass of the CNT with a diameter of 2.5 nm in the *z* direction as a function of time and temperature. Four different temperatures were considered: 300 K, 800 K, 1300 K, and 1800 K. (**b**) Velocity of the center of mass of the CNT in the *z* direction as a function of time at 1800 K. Two CNT diameters were considered: 2.5 nm (red line) and 3.5 nm (black line).

The temperature of the CNT region mimicking the irradiated plasmonic coating is important in determining nanorocket motion. Thus, a series of simulations were conducted to explore the temperature’s influence on CNT velocity. The detailed simulation parameters are outlined in Table 1, with distinct setups denoted as 1, 2, 3, and 4. Velocity transients of the CNT’s center of mass are depicted in Figure 3, illustrating the dependence of velocity on simulated temperature. The results in Figure 3a suggest that the motion of the CNT’s center of mass at 300 K and 800 K is merely characterized by fluctuations, with no significant rocket dynamics observed. On the other hand, the onset of a velocity transient is observed at a temperature of 1300 K. At a temperature of 1800 K the trend is more evident, with distinct acceleration and deceleration phases of the CNT and a maximum velocity of approximately 77 m s^−1^, aligning with the values reported in [22,26,56]. Moreover, the center-of-mass velocity along the *x* and *y* directions exhibits oscillations around zero, which confirms the unidirectional motion of the CNT along the *z* axis. It is worth mentioning that Wei and co-workers [57] asserted the stability of CNTs up to 3400 K through their investigation.

Next, we explored the effect of CNT diameter on velocity. To this end, an additional simulation was performed by considering a CNT with a diameter of 3.5 nm (see setup 5 in Table 1). The heating temperature was set to 1800 K to facilitate the observation of the CNT motion. The results in Figure 3b show that the velocity transients resulting from the CNTs with different diameters exhibited a similar progression of acceleration and deceleration phases. The two CNTs reached a maximum velocity within the range of 70 and 80 m s^−1^, with the 2.5 nm CNT showing a slightly quicker response. The deceleration phase reveals relatively minor differences, being largely affected by thermal fluctuations. Furthermore, maintaining a consistent temperature across both cases (i.e., 1800 K) ensured that equal momentum was carried by individual inner water molecules. It is worth mentioning that a rotational motion around the *z* axis was observed together with the translational one; however, the related rotational kinetic energy was negligible with respect to the translational one, being uncorrelated with the simulated temperature and diameter.

**Figure 4:**
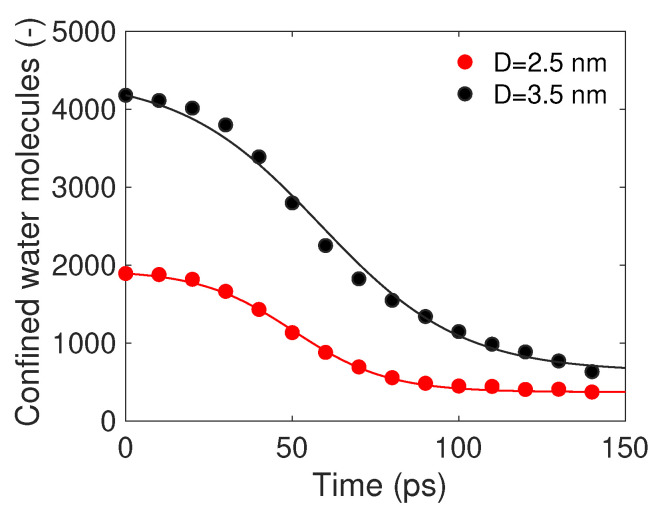
Number of water molecules in two CNTs. The dots represent molecular dynamics simulations and the lines show the fitting curves. Fitting parameters *a* and *b* (refer to Equation (8), R-square > 0.98) are −1.79 and 3.54 × $10^{-2}$ nm^−1^ for the 2.5 nm case and −1.39 and 2.37 × $10^{-2}$ nm^−1^ for the 3.5 nm case, respectively.

For completeness, the number of water molecules that were initially within the CNT and progressively flowed outside after heating it, resulting in propulsion, was accurately assessed as a function of time. The molecular dynamics results are reported in Figure 4; CNTs with a diameter of 2.5 nm (red dots, setup 4) and 3.5 nm (black dots, setup 5) were considered.

The process of water molecule release from within the CNT was observed to initiate gradually, accelerate, and ultimately converge toward an asymptotic constant value. The CNT with 3.5 nm diameter exhibited a relatively faster release of water molecules both at the onset and towards the end of simulation.

### 3.2. Analytical Motion Formulation: Rocket Model

**Figure 5:**
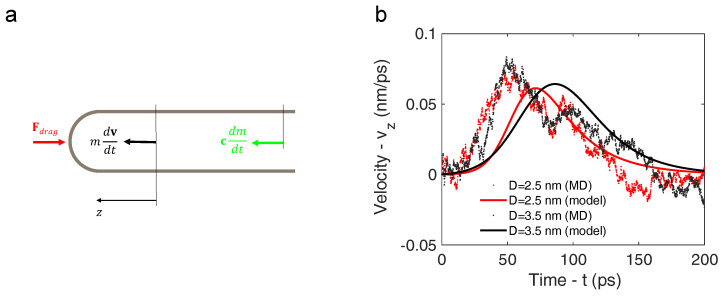
(**a**) Forces acting on the capped nanotube, modeled as a rocket, during its motion. (**b**) Velocities of the CNTs obtained through the simplified rocket model (solid lines) along with velocities obtained through the molecular dynamics simulations (dots). Two CNT diameters were considered: 2.5 nm (red line and dots) and 3.5 nm (black line and dots). The heating temperature was set to 1800 K.

A simplified theoretical rocket model was considered in order to interpret the MD results. The forces exerted on the capped CNT as it moves in a rocket-like fashion are illustrated in Figure 5a. The balance of forces that describes the dynamics of the CNT can be expressed as follows:

(5) $$m\frac{dv}{dt}=c\frac{dm}{dt}+F_{drag}$$

where *m* is the mass of the CNT and confined water, $\frac{dv}{dt}$ is the acceleration of the CNT, c is the velocity vector of water molecules leaving the CNT, and $\frac{dm}{dt}$ is the variation of mass of water molecules confined within the CNT, which will be negative, as the mass in the control volume is reduced with time. We have assumed that **c** is parallel and has a direction opposite to **v**. Finally, $F_{drag}$ represents the drag force calculated according to Stokes’ law:

(6) $$F_{drag}=-6\pi \mu Rv=-K_{drag}v$$

where μ is the dynamic viscosity of water, *R* is the radius of the CNT, v is the velocity of the CNT, and $K_{Drag}=6\pi \mu R$. The use of the Stokes’ law was motivated by the low Reynolds number in the system, which was estimated to be around 0.1. Moreover, as the problem can be safely considered as one-dimensional, Equation (5) can be simplified and written in scalar form:

(7) $$m\frac{dv_{z}}{dt}=-c\frac{dm}{dt}-K_{drag}v_{z}.$$

The mass of confined water molecules within the CNT was modeled using the following fitting equation for the MD results (cf. the solid lines in Figure 4):

(8) $$m\left(t\right)=-\frac{m_{o}-m_{\infty }}{1-\tanh(a)}\tanh\left(bt+a\right)+\frac{m_{o}-m_{\infty }}{1-\tanh(a)}+m_{\infty }$$

where $m_{0}$ is the initial mass, $m_{\infty }$ is the mass asymptotically achieved after the full rocket dynamics (approximately after t=140 ps), and *a* and *b* are fitting parameters. The mass flow rate was found by deriving Equation (8) with respect to the time:

(9) $$\frac{dm}{dt}=-b\frac{m_{0}-m_{\infty }}{1-\tanh(a)}{\left[\mathrm{sech}(bt+a)\right]}^{2}.$$

Then, by invoking the continuity equation with respect to the volume of confinement within the CNT, we obtain

(10) $$c=-\frac{\frac{dm}{dt}}{A\rho },$$

where ρ is the average water density in the CNT and *A* is the cross sectional area of the CNT, which can be computed as follows:

(11) $$A=\frac{\pi D^{2}}{4}$$

where *D* is the diameter of the CNT. Therefore, Equation (7) can be rewritten as

(12) $$\frac{dv_{z}}{dt}=\frac{1}{A\rho m}{\left(\frac{dm}{dt}\right)}^{2}-\frac{K_{drag}}{m}v_{z}.$$

Figure 5 reports the CNT velocities evaluated through Equation (12). In detail, Figure 5b shows the nanotube velocities with diameters equal to 2.5 and 3.5 nm as a function of time, along with the velocities obtained from the molecular dynamics simulations. For simplicity, $K_{drag}$ was estimated by considering the bulk value of viscosity (i.e., 0.1 cP). Nevertheless, the match between the velocity values obtained through the molecular dynamics simulations and those obtained through the simplified model turns out to be acceptable. Moreover, these values are consistent with those reported in [58].

It is fundamental to underline that a simplified rocket model was used to investigate a complex physical phenomenon. Several assumptions were considered, such as employing an average bulk value for $K_{drag}$ and using a continuum approach to describe the intrinsically discrete water transport from the CNT. For instance, Equation (11) assumes that the actual cross-sectional area of the CNT is calculated using the nominal diameter rather than the effective diameter. The impact of CNT’s temperature-induced vibrations on the effective diameter is also unaccounted. On the other side, as a first approximation, the water viscosity was considered as temperature-independent.

Furthermore, it is noteworthy that thermophoretic forces may be induced during the rocket’s motion, exerting an influence on water molecules due to the generated temperature gradient. In the past, a linear model for estimating the velocity induced by a temperature gradient in fullerene (and thus subject to thermophoretic force) has been documented in the literature, e.g., [59]. However, complexities arise as well, as thermophoretic interactions may occur simultaneously with respect to both inner and outer water molecules during the evaporation process. Hence, we opted for a simplified model that disregards any thermophoretic effects while still providing an acceptable description of the phenomenon under investigation.

Indeed, the propulsion mechanism of the nanotube and its duration are critically dependent on the amount of water molecules available for evaporation, which in turn is influenced by the nanotube’s diameter and length. Specifically, larger diameters can accommodate more water molecules, which can prolong the propulsion duration. However, this also increases the drag resistance, which can mitigate some of the propulsion benefits. On the other hand, increasing the CNT’s length directly increases the number of water molecules available for evaporation with a more moderate drag enhancement, thereby potentially extending the propulsion duration more effectively. Additionally, the wetting properties of the nanotube, which can be modified by surface functionalizations, can play a significant role as well; enhanced wetting properties can allow a larger amount of water molecules to enter the nanotube, further influencing the propulsion dynamics [60,61]. In addition, it is important to consider that the process is cyclic and tends to discharge. Therefore, in order to trigger a second propulsion (and thus a more continuous and time-stable process), re-thermalization and refilling of the nanorocket are required.

## 4. Conclusions

We performed molecular dynamics simulations of a system mimicking a gold-coated one-end-capped CNT heated by near-infrared radiation in an aqueous environment. Our simulations demonstrated that phase transitions of water can robustly propel the CNT at exceptionally high velocities, reaching up to 77 m s^−1^. Investigations into different heating temperatures unveiled motion initiation at and beyond 1300 K. Furthermore, we explored two distinct CNT diameters within scaled box sizes, revealing similar velocity evolution patterns and peak velocities across the reported results.

Moreover, we proposed a simplified analytical rocket model to interpret the motion of the heated CNT. Interestingly, the findings from this relatively simple and computationally effective model suggest fair agreement with the results obtained through molecular dynamics simulations. Furthermore, the results call for immediate experimental verification to validate the model and quantify any discrepancies due to CNT functionalization and gold coating. Indeed, the latter may modify the thermal vibrations response of the nanorocket. Furthermore, future investigations could consider other force-fields for the CNT (e.g., AIREBO [62]) that, despite lower computational performance, might represent bonded interactions more accurately. Nevertheless, the observed rocket-like motion is due to nanotube–water heat exchange and the resulting evaporation of water molecules, which are mainly affected by non-bonded interactions.

The implications of designing and realizing nanorockets extend to diverse applications such as drug delivery mechanisms, nanomachines, and robotics, where nanorockets could be tangentially attached to a rotor in cascades, significantly enhancing the rotor’s speed. Furthermore, as the rocket-like motion is mainly due to non-bonded nanotube–water interactions, other types of nanotubes (e.g., made of boron nitride [63], titanium dioxide [64], or silicon [65]) could be potentially employed to experimentally implement the proposed nanorocket concept.

## Figures and Tables

**Table 1:** List of the considered simulation settings and variable parameters. *D* refers to the CNT diameter and *T* to the temperature of the heated region of the capped CNT (cf. *H* in Figure 1c). In all configurations, the length of the CNT was set equal to L=15.7 nm and H=L/2. The number of water molecules refers to the whole computational domain.

Setup	D (nm)	Chirality	Box Size [nm × nm × nm]	T (K)	Water Molecules [-]
1	2.5	19 × 19	8 × 8 × 40	300	83,287
2	2.5	19 × 19	8 × 8 × 40	800	83,287
3	2.5	19 × 19	8 × 8 × 40	1300	83,287
4	2.5	19 × 19	8 × 8 × 40	1800	83,287
5	3.5	27 × 27	12 × 12 × 60	1800	285,769
6	2.5	19 × 19	9 × 9 × 45	1800	119,414

## Data Availability

The raw data supporting the conclusions of this article will be made available by the authors on request.

## Supplementary Materials

The following supporting information can be downloaded at: https://www.mdpi.com/article/10.3390/nano14131134/s1, Video S1: Representative rocket motion for a simulated CNT.
